# Does ROC asymmetry reverse when detecting new stimuli? Reinvestigating whether the retrievability of mnemonic information is task-dependent

**DOI:** 10.3758/s13421-022-01346-7

**Published:** 2022-08-19

**Authors:** Constantin G. Meyer-Grant, Karl Christoph Klauer

**Affiliations:** Department of Psychology, University of Freiburg, 79085 Freiburg, Germany

**Keywords:** Recognition memory, Signal detection theory, ROC asymmetry, Simultaneous detection and identification, Old–new recognition

## Abstract

Recently, it has been suggested that the mnemonic information that underlies recognition decisions changes when participants are asked to indicate whether a test stimulus is new rather than old (Brainerd et al., 2021, *Journal of Experimental Psychology: Learning Memory, and Cognition*, advance online publication). However, some observations that have been interpreted as evidence for this assertion need not be due to mnemonic changes, but may instead be the result of conservative response strategies if the possibility of asymmetric receiver operating characteristics (ROCs) is taken into account. Conversely, recent findings in support of asymmetric ROCs rely on the assumption that the mnemonic information accessed by the decision-maker does not depend on whether an old or a new item is considered to be the target Kellen et al. (2021, *Psychological Review 128*[6], 1022–1050). Here, we aim to clarify whether there is such a difference in accessibility of mnemonic information by applying signal detection theory. To this end, we used two versions of a simultaneous detection and identification task in which we presented participants with two test stimuli at a time. In one version, the old item was the target; in the other, the new item was the target. This allowed us to assess differences in mnemonic information retrieved in the two tasks while taking possible ROC asymmetry into account. Results clearly indicate that there is indeed a difference in the accessibility of mnemonic information as postulated by (Brainerd et al., 2021, *Journal of Experimental Psychology: Learning Memory, and Cognition*, advance online publication).

## Introduction

In the context of recognition memory research, observing changes in the response patterns between experimental conditions raises the important question of how to tease apart the contribution of differences in both response bias (e.g., as the result of certain response strategies) and mnemonic information retrieved by the decision-maker. To address this issue, cognitive models based on *signal detection theory* (SDT; Green & Swets, 1966; Macmillan & Creelman, 2005; Swets, Tanner, & Birdsall, 1961; Wickens, 2002) have long been used to unravel the processes underlying recognition decisions (for a recent overview, see Rotello, 2017; see also Kellen, Winiger, Dunn, & Singmann, 2021).

Such models generally assume that mnemonic stimulus information is mentally represented by a continuous latent memory-strength signal often called *familiarity* (see, e.g., Morrell, Gaitan, & Wixted, 2002; Delay & Wixted, 2021). These familiarity values are stochastic in nature, that is, they are modeled as real-valued random variables (RVs) following a continuous probability distribution. If a test stimulus (e.g., an image) was previously encountered during study, its elicited familiarity value is assumed to be higher on average than the familiarity value of a nonstudied stimulus. Therefore, two familiarity distributions are required: one corresponding to studied (i.e., old) stimuli and the other to nonstudied (i.e., new) stimuli (Macmillan & Creelman, 2005).

Within SDT models of recognition memory, a recognition decision is determined by comparing the familiarity value elicited by the test stimulus with a certain response criterion *λ*. The response given by the decision-maker then corresponds to whether or not the test stimulus’ familiarity value exceeds the response criterion, which results in an “old” or “new” decision, respectively. Thus, the higher (lower) the value of the response criterion, the more conservative (liberal) the response strategy. By assuming a set of ordered response criteria Λ = *λ*_1_,...,*λ*_*k*− 1_ instead of a single response criterion, the model can, furthermore, naturally account for an extended task, in which participants are required to respond on a *k*-level confidence scale (see, e.g., Kellen & Klauer, 2018).

The core theoretic assumption of the SDT model framework, according to which continuously graded memory information is mapped directly onto observable responses, is—in principle—not dependent on any specific parametric form of the old-item and new-item familiarity distributions (Kellen & Klauer, 2018; Kellen et al., 2021; Rouder, Province, Swagman, & Thiele, 2014). Nevertheless, in most applications, such auxiliary assumptions are introduced, mainly for practical reasons. Arguably, the most prominent parametric version of the general SDT framework is the so-called *Gaussian* SDT model, in which familiarity values are assumed to be normally distributed with {*μ*_*o*_,*σ*_*o*_} and {*μ*_*n*_,*σ*_*n*_} being the means and standard deviations of old-item and new-item familiarities, respectively, and *μ*_*o*_ > *μ*_*n*_.^1^

## ROC asymmetry

**Fig. 1:**
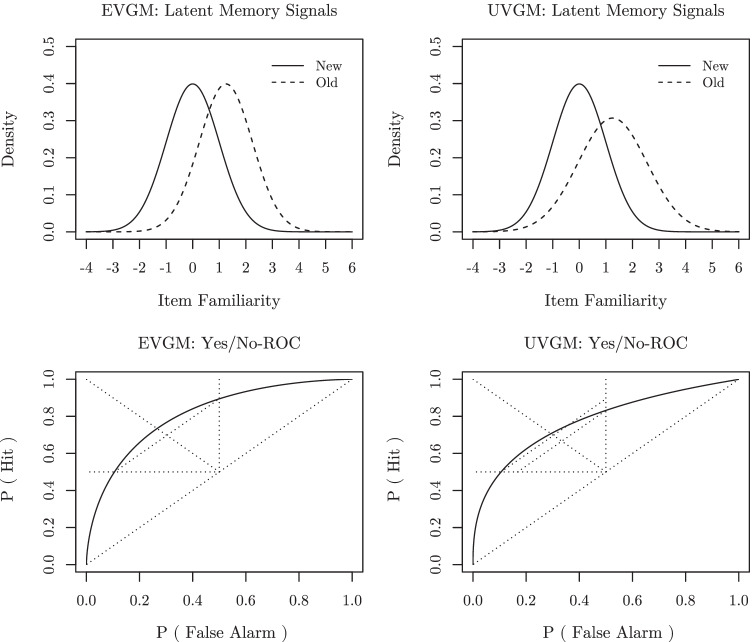
The EVGM (*left column of panels*) with parameters *μ*_*o*_ = 1.25, *μ*_*n*_ = 0.00, and *σ*_*o*_ = *σ*_*n*_ = 1.00 and the UVGM (*right column of panels*) with parameters *μ*_*o*_ = 1.25, *μ*_*n*_ = 0.00, *σ*_*o*_ = 1.30, and *σ*_*n*_ = 1.00. *Top row of panels* depicts the probability density functions of old-item (*dashed lines*) and new-item (*solid lines*) familiarity distributions, and the *bottom row of panels* depicts the corresponding ROCs for a single-item yes/no recognition task. Note that the ROC of the EVGM depicted in the bottom left panel is symmetric (i.e., it contains both the points {P(Hit), P(False Alarm)} and {1 − P(Hit), 1 − P(False Alarm)}; see also Kellen et al., 2021; Killeen & Taylor, 2004), whereas the ROC of the UVGM depicted in the bottom right panel is not

When plotting the predicted probabilities of a *hit* (“old” responses to old items) and a *false alarm* (“old” responses to new items) against each other for different response criteria, while holding the underlying old-item and new-item familiarity distributions constant, the resulting curve is referred to as the predicted *receiver operating characteristic* (ROC; Macmillan & Creelman, 2005; Kellen & Klauer, 2018; Yonelinas & Parks, 2007). Importantly, an *equal-variance Gaussian model* (EVGM)—a special case of the Gaussian SDT model that assumes *σ*_*o*_ = *σ*_*n*_—predicts symmetric ROCs (Killeen & Taylor, 2004). But a consistent finding in recognition memory tasks is that empirical ROCs based on observed relative response frequencies are asymmetric (see, e.g., Yonelinas, 1994; Ratcliff, Sheu, & Gronlund, 1992; Glanzer, Kim, Hilford, & Adams, 1999; Egan, 1958; Dubé & Rotello, 2012; Yonelinas & Parks, 2007). More precisely, relative to predictions derived from models with symmetric ROCs, conservative responses are associated with more hits than one would expect based on the relative frequency of false alarms and vice versa for liberal responses (see Fig. 1).

To account for this observation, the *unequal-variance Gaussian model* (UVGM) assumes not only that *μ*_*o*_ > *μ*_*n*_, but also that *σ*_*o*_ > *σ*_*n*_ (see, e.g., Ratcliff et al., 1992; Rotello, 2017; Jang, Wixted, & Huber, 2009), where we can further set *σ*_*n*_ = 1 and *μ*_*n*_ = 0 without loss of generality. Figure 1 illustrates how differences between the variance of old-item and new-item familiarity distributions lead to differences in ROC asymmetry within the Gaussian SDT model.

Recently, Kellen et al. (2021, see Experiment 3) investigated ROC asymmetry by means of two different recognition memory tasks, namely an *m-alternative forced-choice* task and what they called an m*-alternative forced-choice task. In the latter task, a single new stimulus is presented along *m* − 1 old stimuli and the decision-maker is tasked with identifying the new stimulus (see also Iverson & Bamber, 1997). In contrast, the old stimulus has to be identified among *m* − 1 new stimuli in the standard *m*-alternative forced-choice task. Interestingly, if the memory-strength distributions give rise to asymmetric ROCs, identification performance is predicted to differ between the m*-alternative and the *m*-alternative forced-choice task if *m* > 2, whereas identification performance is predicted to be the same in both tasks if ROCs are symmetric. Thus, comparing correct identification rates (see Kellen et al., 2021, Experiment 3) in both the m*-alternative and *m*-alternative forced-choice responses enabled them to conduct a distribution-free test of ROC asymmetry without relying on confidence judgments or selective manipulation of response criteria (i.e., bias manipulation). Their results corroborated the notion of ROC asymmetry as usually observed in standard recognition memory paradigms (Kellen et al., 2021).

This conclusion, however, critically depends on the assumption that the same latent memory-strength distributions underlie both tasks, but recent findings have cast some doubt on whether this assumption in fact holds (Brainerd, Bialer, Chang, & Upadhyay, 2021). Based on data obtained via *single-item yes/no recognition* tasks, Brainerd et al. (2021) have argued that fundamentally different mnemonic information may be accessed by participants when they are asked to decide whether or not an item is new (i.e., detecting *newness*) instead of whether or not an item is old (i.e., detecting *oldness*).

Brainerd et al. (2021) observed, for example, that the relative frequency of correct responses for old items was greater when participants were asked to detect whether the current stimulus is new compared to when they were asked to detect whether it is old. Conversely, they also found that the relative frequency of correct responses for new items was greater when participants were asked to detect whether the current stimulus is old compared to when they were asked to detect whether it is new. Although these effects are not difficult to explain solely by means of certain response strategies (i.e., by the placement of the response criterion)—participants may simply tend to answer conservatively, that is, they try to avoid false alarms in both cases (i.e., answering “new” to an old item when asked whether the item is old, or answering “old” to a new item when asked whether the item is new)—the results also suggested that overall detection performance was better when detecting oldness than when detecting newness.

This was substantiated, inter alia, by fitting a Gaussian SDT model to their data, which revealed (along with differences in response bias) that the difference between *μ*_*n*_ and *μ*_*o*_ was systematically smaller in cases where participants were asked to detect newness compared to when they were asked to detect oldness. Brainerd et al. (2021) attributed these effects to changes in accessibility and activation of certain memory traces in terms of *fuzzy-trace theory* (see also Brainerd, Nakamura, & Lee, 2019; Brainerd & Reyna, 2005; Brainerd & Reyna, 2008). In other words, they hypothesized that the mnemonic information underlying the respective recognition decision differs between different situations, that is, between different combinations of stimulus type (i.e., old vs. new) and task (i.e., detecting oldness vs. newness).

Notably, however, Brainerd et al. (2021) could only estimate the parameters of an EVGM, which is—as mentioned previously—unable to account for ROC asymmetry. This is unfortunate, as we will see in the following that in the case of asymmetric ROCs, some qualitative response patterns observed by Brainerd et al. (2021) are in fact also consistent with the absence of differences in mnemonic information underlying the recognition decision in both tasks.

## SDAI and SDAI* tasks

In the present work, we aim to reinvestigate both ROC asymmetry and whether there is a difference in retrieved mnemonic information between detecting newness and oldness by combining the approaches by Kellen et al. (2021) and Brainerd et al. (2021) with the so-called *simultaneous detection and identification* (SDAI; Macmillan & Creelman, 2005) compound task. This task is well known by researchers of eyewitness identification, as it is akin to the simultaneous lineup procedure (Mickes & Gronlund, 2017; Gronlund & Benjamin, 2018), but it was, for instance, also recently used by Meyer-Grant and Klauer (2021) to evaluate different models of recognition memory. In essence, it is comprised of two distinct—but closely related—sub-tasks that arise when, among a set of *m* stimuli, a *target* (usually an old item) is either present or not. This situation requires a decision-maker to decide, first, if a target is present (a target trial) or absent (a non-target trial; i.e., all presented stimuli are *lures*) in the current set of stimuli and, second, which of the currently presented stimuli is most likely to be the target. The first sub-task is usually referred to as *1-out-of-m* *detection*, while the second sub-tasks correspond to an *m*-alternative forced-choice identification task.

SDAI allows to derive ROCs from the responses in the detection sub-task by plotting the relative frequencies of correctly detecting the presence of a target in a target trial (i.e., the frequencies of hits in the detection sub-task) against the relative frequencies of falsely detecting the presence of target in a non-target trial (i.e., the frequencies of false alarms in the detection sub-task).^2^ The identification responses, on the other hand, give rise to the so-called *identification operating characteristic* (IOC; Macmillan & Creelman, 2005), which plots the relative frequency of a hit in the detection sub-task and a correct subsequent identification of the target against the relative frequency of a false alarm in the detection sub-task.

The same basic idea utilized in Kellen et al.’s (2021) Experiment 3 as well as in Brainerd et al.’s (2021) Experiments can be implemented in an SDAI task. That is, one can instruct participants to detect and identify a new stimulus instead of an old one. Thus, a new stimulus becomes the target in this setting while old stimuli can be considered lures. In order to correspond to the notation of Kellen et al. (2021), we refer to this new compound task as SDAI* (comprised of both the 1-out-of-m* *detection* and the m*-alternative forced-choice identification sub-tasks) in the following.^3^ This approach is interesting in that it not only provides us with correct identification rates, but also allows us to construct empirical ROCs for both tasks by combining it, for example, with a confidence rating approach.

**Fig. 2:**
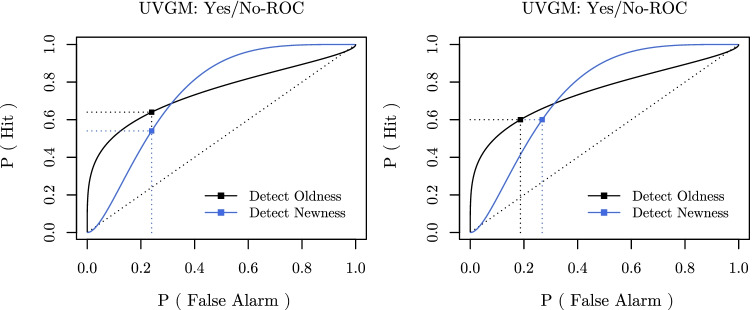
ROCs of an UVGM with parameters *μ*_*o*_ = 1.33, *μ*_*n*_ = 0.00, *σ*_*o*_ = 1.74, and *σ*_*n*_ = 1.00 for a single-item yes/no recognition task in which either the old item is the target (*black*) or in which the new item is the target (*blue*). *Left panel*: *dotted lines* and *squares* indicate the predicted hit rates for either detecting oldness (0.64; *black*) or newness (0.54; *blue*) when the predicted false alarm rate (0.24) remains constant. *Right panel*: *dotted lines* and *squares* indicate the predicted false-alarm rates for either detecting oldness (0.19; *black*) or newness (0.27; *blue*) when the predicted hit rate (0.60) remains constant

Importantly, ROC asymmetry reverses when the task is to detect newness instead of oldness for models that predict asymmetric ROCs in the first place (as, e.g., the UVGM), which also holds for single-item yes/no recognition tasks. This is illustrated in Fig. 2, which depicts the yes/no-ROCs for both cases.^4^ Assuming the type of ROC asymmetry typically observed in recognition memory research (see, e.g., Ratcliff et al., 1992; Wixted, 2007; Kellen et al., 2021), this implies, for instance, that for a given false alarm rate to the left of the intersection of the two ROC curves (i.e., for a more or less conservative response criterion), hit rates will be larger when detecting oldness than when detecting newness (see Fig. 2, left panel). For the same reason, achieving the same hit rate when detecting newness instead of oldness will be accompanied by a higher false alarm rate for relatively conservative response criteria (see Fig. 2, right panel). Given that a decision-maker tends to avoid false alarms, it is thus to be expected that performance in detecting newness appears to be worse compared to performance in detecting oldness (i.e., a lower observed hit rate and/or a higher observed false alarm rate).

Interestingly, these effects seem to match certain results reported by Brainerd et al. (2021). Taking a look at their experimental data reveals, for example, that in those instances where the false alarm rate was approximately constant between the two tasks (i.e., detecting oldness vs. newness), the hit rate was lower when participants were asked to detect newness than when they were asked to detect oldness (see, e.g., data pooled over the initial tests in Experiments 5–7 in Brainerd et al., 2021, p. 9, where the hit rate for detecting oldness and newness was reported to be 0.64 and 0.54, respectively, whereas the false alarm rate in both cases was 0.24; see also Fig. 2, left panel). Hence, ROC asymmetry appears to be a plausible alternative explanation for some of the findings by Brainerd et al. (2021), including decreased overall performance when detecting newness.

However, this raises the question of whether assuming a modulation of the mnemonic information underlying recognition decisions (Brainerd et al., 2021) is necessary when allowing for asymmetric ROCs. Moreover, addressing this question is critical not only for investigating mnemonic differences between tasks that focus on detecting oldness versus newness, but also—as mentioned earlier—for other studies investigating ROC asymmetry that have been based on the assumption that such differences do not exist (Kellen et al., 2021). Fortunately, having participants complete both the SDAI and the SDAI* task allows us to assess the mnemonic consistency of models across tasks, while taking potential ROC asymmetry into account.

Thus, by jointly investigating both SDAI and SDAI* we pursue three main research objectives that correspond to the three following questions:

1. Does ROC asymmetry in an SDAI* task in fact reverse compared to ROC asymmetry in an SDAI task?
2. Can both SDAI and SDAI* be modeled by an UVGM (which allows for asymmetric ROCs) with constant mnemonic parameters across tasks?
3. Can both SDAI and SDAI* be modeled by any model belonging to the general nonparametric SDT model framework with constant latent memory-strength distributions across tasks?

In what follows, we outline how these questions will be addressed and answered. To this end, however, it is first necessary to provide a brief overview of how both SDAI and SDAI* are modeled within the SDT model framework.

### SDT models of SDAI and SDAI* tasks

SDT models for SDAI have been known for quite some time in the SDT literature (Starr, Metz, Lusted, & Goodenough, 1975; Green & Birdsall, 1978; Macmillan & Creelman, 2005; Meyer-Grant & Klauer, 2021; Wixted & Mickes, 2014; Wixted, Vul, Mickes, & Wilson, 2018). According to such models, a separate familiarity value is elicited by each of the simultaneously presented test stimuli. Since in the following we will focus on situations in which the stimuli of a set do not resemble each other systematically, it is reasonable to assume that these familiarity values are independent RVs (see Meyer-Grant & Klauer, 2022), which follow either the old-item or new-item familiarity distribution, depending on whether the corresponding stimulus is old or new.

We denote the probability density function (PDF) and cumulative distribution function (CDF) as *f*_*o*_(⋅) and *F*_*o*_(⋅), respectively, for an old item and as *f*_*n*_(⋅) and *F*_*n*_(⋅), respectively, for a new item. If the maximum of all simultaneously elicited familiarity values exceeds the response criterion, a ”target presence” response is given, whereas otherwise a “target absence” response is given. Hence, the probability of a hit in the 1-out-of-*m* detection sub-task (H; i.e., correctly detecting the presence of an old item) is given by

$$\mathrm{P}_{\text{SDT}} (\mathrm{H}) = 1 - F_{o}(\lambda ) [F_{n}(\lambda )]^{m-1} ,$$

since the complementary event is that none of the *m* familiarity values exceeds the response criterion *λ*.^5^ Following a similar logic as for the probability of a hit, the probability of a false alarm in the 1-out-of-*m* detection sub-task (FA; i.e., incorrectly detecting the presence of an old stimulus) is given by

$$\mathrm{P}_{\text{SDT}} (\text{FA}) = 1 - [ F_{n}(\lambda ) ]^{m} .$$

The *m*-alternative forced-choice identification response, on the other hand, is determined by which stimulus elicited the highest familiarity value, which is why the joint probability of a hit in the 1-out-of-*m* detection sub-task and a subsequent correct identification in the *m*-alternative forced-choice sub-task (I; i.e., identification of the old stimulus) is given by

1 $$\mathrm{P}_{\text{SDT}} (\text{I,H}) = {\int}_{\lambda}^{\infty} [F_{n}(x)]^{m-1}f_{o}(x)\mathrm{d}x .$$

To get an intuition for Eq. 1, first consider that integrating the PDF of the old-item familiarity distribution *f*_*o*_(*x*) over the interval $(\lambda , \infty )$ yields the probability that the old-item familiarity value exceeds *λ*. However, if for each potential old-item familiarity value $x \in (\lambda , \infty )$ we additionally scale down *f*_*o*_(*x*) by the probability that all *m* − 1 simultaneously elicited new-item familiarity values fall below *x* (note that this probability is given by [*F*_*n*_(*x*)]^*m*− 1^ ≤ 1; see also Footnote 5), integrating over the interval $(\lambda , \infty )$ instead yields the joint probability of the old-item familiarity exceeding both *λ* and all new-item familiarities.

In order to account for SDAI* instead of SDAI, this model framework can be adapted without much difficulty: Crucially, it is no longer the maximum (as in the 1-out-of-*m*-detection sub-task) but the minimum of all familiarity values that determines the 1-out-of-m*-detection response. If it falls below the response criterion, a ”target present” response is given, while otherwise a ”target absence” response is given.^6^ Hence, the probability of a hit in the 1-out-of-m* detection sub-task (H*; i.e., correctly detecting the presence of a new stimulus) is given by

$$\mathrm{P}_{\text{SDT}} (\mathrm{H^{*}}) = 1 - \left([1 - F_{o}(\lambda )]^{m-1}[1 - F_{n}(\lambda )]\right) ,$$

since the complementary event is that all of the *m* familiarity values exceed the response criterion *λ*.^7^ The probability of a false alarm in the 1-out-of-m* detection sub-task (FA*; i.e., incorrectly detecting the presence of a new stimulus) is in turn given by

$$\mathrm{P}_{\text{SDT}} (\text{FA}^{*}) = 1 - [1 - F_{o}(\lambda ) ]^{m} .$$

The m*-alternative forced-choice identification response is then consequently determined by which stimulus elicited the lowest familiarity value and the joint probability of a hit in the 1-out-of-m* detection sub-task and a subsequent correct identification in the m*-alternative forced-choice sub-task (I*; i.e., identification of the new stimulus) is thus given by

2 $$\mathrm{P}_{\text{SDT}} (\mathrm{I}^{*},\mathrm{H}^{*}) = {\int}_{-\infty}^{\lambda} [1-F_{o}(x)]^{m-1}f_{n}(x)\mathrm{d}x .$$

In contrast to Eq. 1, in Eq. 2 we scale down the PDF of the new-item familiarity distribution *f*_*n*_(*x*) for each potential new-item familiarity value *x* that falls below the response criterion *λ* (i.e., $x \in (-\infty , \lambda )$) according to the probability that all the simultaneously elicited old-item familiarity values exceed *x* (note that this probability is given by [1 − *F*_*o*_(*x*)]^*m*− 1^ ≤ 1; see also Footnote 5 and 7). Evaluating the integral in Eq. 2 thus corresponds to the joint probability that the new-item familiarity falls below both *λ* and all old-item familiarities.

**Fig. 3:**
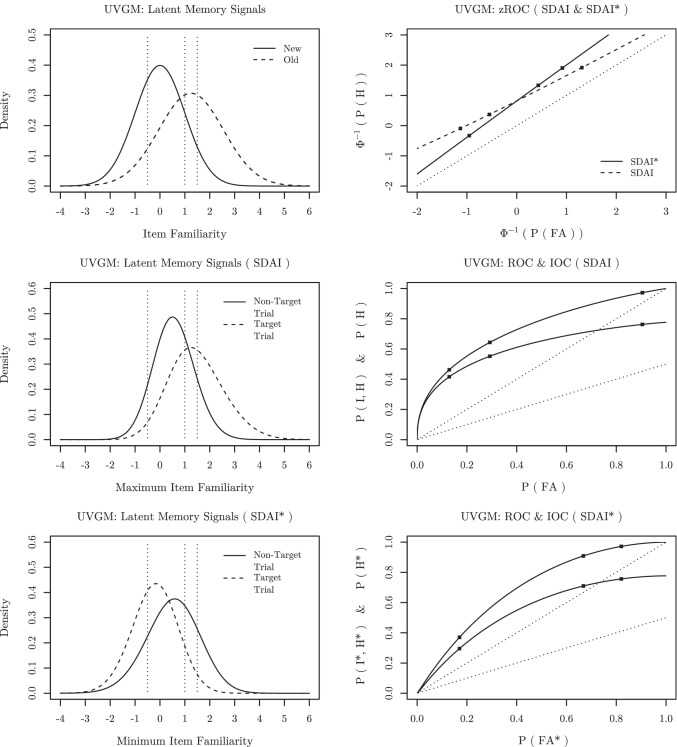
Illustration of the UVGM with parameters *μ*_*o*_ = 1.25, *μ*_*n*_ = 0.00, *σ*_*o*_ = 1.30, *σ*_*n*_ = 1.00 for both the SDAI and SDAI* task with *m* = 2 simultaneously presented test stimuli. *Dotted vertical lines* in the left column of panels indicate the positions of the response criteria Λ = {− 0.5,1,1.5} and *black squares* in the right column of panels indicate the corresponding predicted response frequencies. *Top left panel*: PDFs of old-item (*dashed line*) and new-item (*solid line*) familiarity distributions. *Middle left panel*: PDFs of the maximum familiarity value (i.e., the decision variable in an SDAI task) for a trial with one old and one new item (i.e., an SDAI target trial; *dashed line*) and for a trial with two new items (i.e., an SDAI non-target trial; *solid line*). *Bottom left panel*: PDFs of the minimum familiarity value (i.e., the decision variable in an SDAI* task) for a trial with one new and one old item (i.e., an SDAI* target trial; *dashed line*) and for a trial with two old items (i.e., an SDAI* non-target trial;*solid line*). *Top right panel*: zROCs for both the SDAI (*dashed lines*) and the SDAI* task (*solid lines*). *Middle right panel*: ROC (*upper line*) and IOC (*lower line*) for the SDAI task. *Bottom right panel*: ROC (*upper line*) and IOC (*lower line*) for the SDAI* task

For illustration purposes, Fig. 3 depicts the PDFs of the old-item and new-item familiarity distributions for the UVGM, as well as the respective PDFs of the maximum familiarity values in SDAI tasks and the minimum familiarity values in SDAI* tasks (both with *m* = 2). Furthermore, Fig. 3 also depicts the corresponding ROCs and IOCs for both tasks.

### Testing for changes in ROC asymmetry

In order to investigate ROC asymmetry, it is helpful to transform the ROC space by applying the quantile function of a standard normal distribution Φ^− 1^(⋅). This gives rise to the so-called zROC. In a standard yes/no recognition task, where a single stimulus at a time is judged to be old or new, this leads to a linear zROC for the Gaussian SDT model. Moreover, the slope of this zROC corresponds to the ratio of standard deviations of the old-item and the new-item familiarity distribution. Strictly speaking, this theoretical justification does not apply for an SDAI and SDAI* task even if the normality assumption holds.^8^ However, the (approximate) slope of the zROC still provides a good indication for the quality of ROC asymmetry. For reasonable parameters, the UVGM, for example, consistently predicts approximate zROC slopes below one for the SDAI task and above one for the SDAI* task (see Fig. 3).^9^ We can therefore evaluate ROC asymmetry by comparing empirical zROC slopes of the SDAI and SDAI* tasks by means of an ordinary least squares linear regression.

### Evaluating the mnemonic consistency of the UVGM

Given that a qualitative change in ROC asymmetry can be observed, it should be investigated whether the UVGM can model both tasks without changes in the familiarity distribution of old items. It could be argued that the response criteria Λ are at least partially under the volitional control of the decision-maker and therefore may vary task-dependently. The difference between the means *μ*_*o*_ − *μ*_*n*_ = *μ*_*o*_ and the ratio of the standard deviations *σ*_*o*_ / *σ*_*n*_ = *σ*_*o*_ of the old-item and new-item familiarity distributions, on the other hand, should not differ systematically between both tasks if the study phases are identical and the assumption holds that the mnemonic information accessed by the decision-maker does not change depending on the task. Contrary to this, however, Brainerd et al. (2021) posited—as mentioned earlier—that the mnemonic information does in fact differ between tasks that ask for detecting oldness and tasks that ask for detecting newness.

To test these competing accounts, we can fit the UVGM model using a maximum likelihood approach to both the SDAI and the SDAI* task twice: once by estimating the parameters of both tasks separately and another time by fitting a joint model of both tasks, were only one *μ*_*o*_ and one *σ*_*o*_ parameter are estimated. If the goodness-of-fit deteriorates significantly for the joint modeling approach compared to the separate one, this indicates mnemonic inconsistencies within the UVGM between the SDAI and the SDAI* task. In particular, this would also challenge our alternative explanation of some of the major results reported by Brainerd et al. (2021), according to which more or less conservative response criteria in combination with ROC asymmetry could be responsible for observed differences between the two tasks.

### Evaluating the mnemonic consistency of the nonparametric SDT model

However, fundamental issues with the UVGM have long been known: most notably, the so-called *likelihood ratio monotonicity* does not hold for the UVGM (Kellen & Klauer, 2018; Green & Swets, 1966; Kellen et al., 2021; Meyer-Grant & Klauer, 2021). A critical consequence of this circumstance is that very low familiarity values are more likely for old than for new stimuli, which is universally considered implausible. The results of the UVGM-based analyses therefore may depend on potentially invalid auxiliary assumptions. Hence, should our analyses indeed reveal mnemonic inconsistencies within the UVGM, the question remains whether the observed patterns can be accounted for by any other task-independent SDT model (i.e., when allowing for arbitrarily distributed familiarity values).

In order to investigate this question, let us once more consider the findings of Kellen et al. (2021), who showed that a symmetric ROC implies that the correct identification probabilities in the *m*-alternative forced-choice and m*-alternative forced-choice sub-tasks must be equal when the latent memory-strength distribution of old items remains unchanged between the tasks. But in contrast to this, they observed that m*-alternative forced-choice correct-rates were consistently below *m*-alternative forced-choice correct-rates for *m* ∈{4,5,6}, which they interpreted as evidence in favor of ROC asymmetry. However, the same result would have been observed under distributions predicting symmetric ROCs if those distributions had changed between tasks (e.g., a decline in the *μ*_*o*_ parameter of an EVGM if participants are asked to identify a new instead of an old item).

Interestingly, choosing *m* = 2 leads to conflicting predictions between these possible scenarios, enabling us to test them directly. In contrast to the cases with *m* > 2 (as investigated by Kellen et al., 2021), the probabilities for a correct identification in the *m*-alternative forced-choice and m*-alternative forced-choice sub-tasks (i.e., the probability of the old-item familiarity being larger than the maximum of new-item familiarities and the new-item familiarity being smaller than the minimum of old-item familiarities, respectively) must be equal in cases with *m* = 2 and task-invariant distributions of familiarity values, regardless of whether or not there is ROC asymmetry.^10^ If, on the other hand, a change in the underlying memory-strength distribution between the two tasks were responsible for the lower m*-alternative forced-choice correct-rates compared to the *m*-alternative forced-choice correct-rates observed by Kellen et al. (2021) for *m* ∈{4,5,6}, the same pattern should be observable for *m* = 2 as well. Thus, simultaneously presenting *m* = 2 stimuli during each test trial and comparing the *m*-alternative forced-choice identification performance in target trials of an SDAI task with the m*-alternative forced-choice identification performance in target trials of an SDAI* task provides a critical test of these conflicting predictions.

## Methods

To investigate these issues, we conducted an experiment in which participants had to complete both an SDAI task and an SDAI* task (for both tasks, *m* = 2 stimuli were presented simultaneously in each test trial). Therefore, each participant took part in two sessions, which were separated by at least one week. One half of the participants were given the SDAI task on their first appointment and the SDAI* task on their second appointment, and vice versa for the other half of participants.

### Participants

Forty-eight participants (39 females, 9 males) aged between 18 and 44 (*M*_*age*_ = 22.79, *SD*_*age*_ = 4.70) completed both experimental sessions. In exchange for their participation, they received either partial course credit or €6.00. Additionally, each participant received a performance-based bonus of up to €3.00. All participants were native or fluent speakers of German and had normal or corrected to normal vision and no prosopagnosia.

### Stimuli and apparatus

We used 1250 color portrait images (depicting 625 females and 625 males), which were all generated by a generative adversarial network (Karras, Laine, & Aila, 2019). All images were crosschecked for image artifacts and a believable appearance by a human rater, who was naïve to the objective of the study.

Each image had a resolution of 250 px × 300 px and was presented on a 522 mm × 294 mm TFT-LCD screen with a resolution of 1920 px × 1080 px. Viewed from a distance of approximately 600 mm, they subtended an angle of about $6^{\circ }29^{\prime }24^{\prime \prime } \times 7^{\circ }46^{\prime }48^{\prime \prime}$. The images were presented on a black background.

### Design and procedure

Both parts of the experiment (viz., the SDAI and the SDAI* task) comprised a study phase and a test phase. The procedure of the study phase was identical for both parts, but different stimuli were shown in each part. Each study phase comprised two blocks of 154 individual portrait images (308 images in total per part). Participants were asked to memorize the images, which were presented successively for 2000 ms each with an interstimulus interval of 800 ms. Between the two blocks, participants were allowed to take a self-paced break. The first and last two images shown during each block of the study phase (eight images in total) were not used during the test phase to mitigate primacy and recency effects. After the study phase, there was a mandatory break of 5 min. After this break, the participants had to solve a short arithmetic problem before continuing with the test phase.

The test phase for both experimental parts comprised 200 trials, respectively, which were evenly divided into four blocks of 50 trials. The blocks were separated by self-paced breaks. In each test trial, participants were shown *m* = 2 same-sex portrait images arranged horizontally (side by side) in the center of the screen. For the SDAI task, two new stimuli were presented during half of the trials (i.e., non-target trials), while an old stimulus was presented together with a new stimulus during the other half of the trials (i.e., target trials). For the SDAI* task, on the other hand, two old stimuli were presented during half of the trials (i.e., non-target trials), while a new stimulus was presented together with an old stimulus during the other half of the trials (i.e., target trials). This resulted in 300 new images being presented in the test phase of the SDAI task and 100 new images being presented in the test phase of the SDAI* task, in addition to the 100 old images for the SDAI task and the 300 old images for the SDAI* task already presented in the respective study phase. Thus, apart from the primacy and recency buffers, all stimuli presented during the study phase reappeared during the test phase of the SDAI* task to serve as lures. In contrast, only one-third of all studied images were randomly selected to serve as targets in the SDAI task. The position of the target (left vs. right) was randomly determined for each target trial with the constraint that the frequency of targets appearing on the left was the same as that of targets appearing on the right across all trials of the same session.

For each of the two experimental parts and each participant, the stimuli—608 for the SDAI task and 408 for the SDAI*—were randomly drawn without replacement from the pool of the 1250 portrait images with the constraint that there was an equal proportion of male and female faces. Which images were presented during study (i.e., old stimuli) and which only appeared during test (i.e., new stimuli), was likewise randomized for each participant, again ensuring an equal proportion of male and female faces for old as well as new stimuli.

Participants were informed prior to the test phase that there would either be one or no target present in each trial and that target and non-target trials would occur in equal frequency. They were further instructed that in the SDAI task, old stimuli should be considered as targets, whereas in the STAI* task, new stimuli should be considered as targets.

Participants were first asked to provide a four-level confidence rating (4 = “target definitely present”, 3 = “target likely present”, 2 = “target likely absent”, or 1 = “target definitely absent”) on whether they believed a target to be present or not. These response options were presented at the bottom of the screen together with the images and participants responded by selecting one of these options with the computer mouse. Subsequently, they were required to identify the image which they believed to be most likely the target, irrespective of their previous confidence rating response.^11^ They indicated their decision by clicking on one of the two images (again, with the computer mouse).

Prior to the start of the experiment, participants were also informed that their final payment would be partly based on their performance in the test phase. That is, participants received a point for a correct detection response (i.e., a “target definitely present” or “target likely present” response in target trials or a “target likely absent” or “target definitely absent” response in a non-target trials). They received an additional point for each correct identification response (i.e., clicking on a target image). Participants were awarded €0.01 for each point they scored above the 300-point mark, up to a maximum of €3.00. No feedback was given during the experiment, but the final point score for each experimental part was presented after their respective completion.

## Results

### Differences in ROC asymmetry

**Fig. 4:**
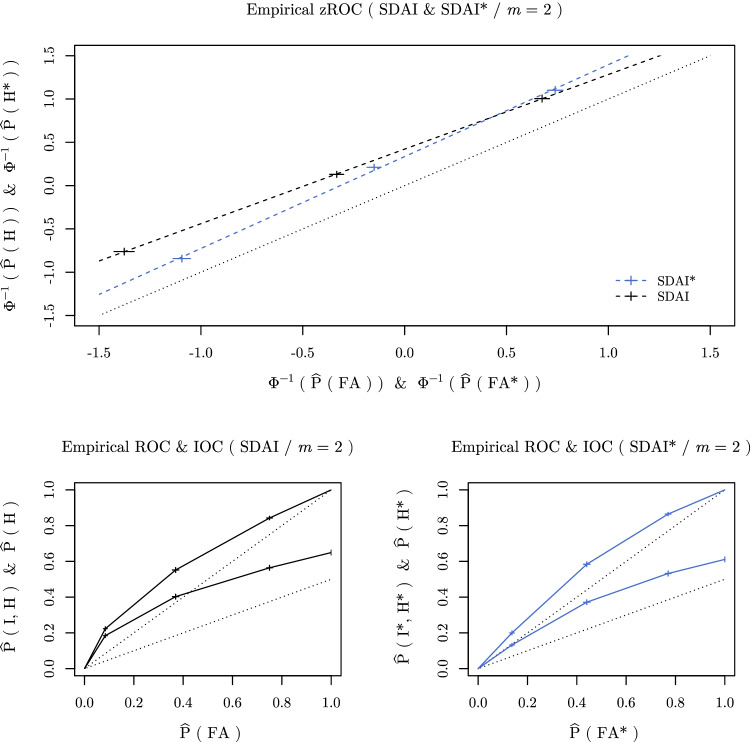
Relative response frequencies (*crosses*) in the data aggregated across participants. The length of the *cross lines* correspond to 95% bootstrap CIs. *Top panel*: empirical zROCs and the corresponding ordinary least squares linear regression lines (*dashed lines*) for both the SDAI (*black*) and the SDAI* (*blue*) tasks. *Bottom row of panels*: empirical ROCs (*upper line*) and IOCs (*lower line*) for both the SDAI (*left panel*, *black*) and the SDAI* (*right panel*, *blue*) tasks

We first computed empirical zROC points for the data aggregated across participants separately for the SDAI as well as the SDAI* task. The ordinary least squares linear fit for the SDAI tasks (regressing ${\Phi }^{-1}(\widehat {\mathrm {P}}(\mathrm {H}))$ on ${\Phi }^{-1}(\widehat {\mathrm {P}}(\text {FA}))$) revealed an approximate zROC slope of 0.86, while the ordinary least squares linear fit for the SDAI* tasks (regressing ${\Phi }^{-1}(\widehat {\mathrm {P}}(\mathrm {H}^{*}))$ on ${\Phi }^{-1}(\widehat {\mathrm {P}}(\text {FA}^{*}))$) revealed an approximate zROC slope of 1.06 (for a pictorial representation see Fig. 4). We then repeated this analysis for each participant individually (note that one participant’s data did not permit estimating the zROC slope for the SDAI* task due to empty cells). The mean of the approximate individual zROC slopes was 0.86 (95% CI [0.81, 0.91]) for the SDAI task and 1.07 (95% CI [1.02, 1.12]) for the SDAI* task. A paired *t* test revealed that the mean difference of 0.21 (95% CI [0.15, 0.28]) between the approximate zROC slopes of the SDAI task and the SDAI* task is significant (*t*(46) = 7.01, *p* < .001).

### Mnemonic consistency of the UVGM

**Fig. 5:**
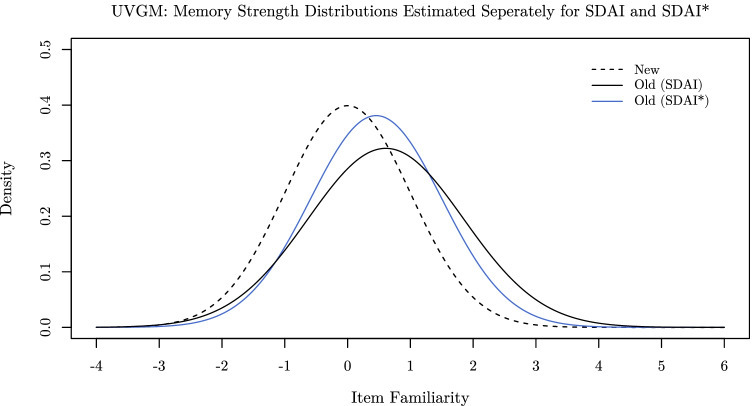
PDFs of old-item (*solid lines*) and new-item (*dashed line*) familiarity distributions according to two separate UVGMs, which were fitted to the data (aggregated across participants) from both the SDAI task (*μ*_*o*_ = 0.61 and *σ*_*o*_ = 1.24; *black solid line*) and the SDAI* task (*μ*_*o*_ = 0.46 and *σ*_*o*_ = 1.05; *blue solid line*), respectively

Next, we fitted two specific UVGM models to the data. One model was essentially equivalent to two separate UVGMs which were fitted to the data of one of the two tasks, respectively, whereas the other model restricted the parameters *μ*_*o*_ and *σ*_*o*_ to be identical for both tasks, while the response criteria Λ were allowed to vary between them. Since these models are clearly nested—the restricted model being a special case of the unrestricted model—we can simply compare them by means of a likelihood-ratio test. Doing so revealed a clear impairment of goodness-of-fit if *μ*_*o*_ and *σ*_*o*_ are restricted to be identical for both SDAI and SDAI* tasks ($\chi _{LR}^{2}(2) = 46.64$, *p* < .001). If left unrestricted, *μ*_*o*_ and *σ*_*o*_ are both estimated to be larger in the SDAI task (*μ*_*o*_ = 0.61 and *σ*_*o*_ = 1.24) compared to the SDAI* task (*μ*_*o*_ = 0.46 and *σ*_*o*_ = 1.05), as depicted in Fig. 5.

However, it is well known that aggregating data across participants can be problematic, as this practice relies on the unrealistic assumption that the model parameters are identical for all participants. We therefore fitted both variants of the UVGM to the data of each participant separately. Since the likelihood-ratio test statistic is assumed to be asymptotically *χ*^2^ distributed, we aggregated the individual test statistics to obtain a measure for overall goodness-of-fit. Results coincide with the analysis of aggregated data in that they reveal a significantly worse fit of the restricted model compared to the unrestricted one ($\chi _{LR}^{2}(96) = 321.45$, *p* < .001). Moreover, two paired *t* tests corroborate the systematic nature of differences in the parameter estimates of *μ*_*o*_ ($M_{\mathrm {diff.} (\mu _{o})} = 0.13$, 95% CI [0.02, 0.24], *t*(47) = 2.36, *p* = .022) and *σ*_*o*_ ($M_{\text {diff.} (\sigma _{o})} = 0.20$, 95% CI [0.09, 0.32], *t*(47) = 3.60, *p* < .001) between the SDAI and the SDAI* task.

### Mnemonic consistency of the nonparametric SDT model

**Fig. 6:**
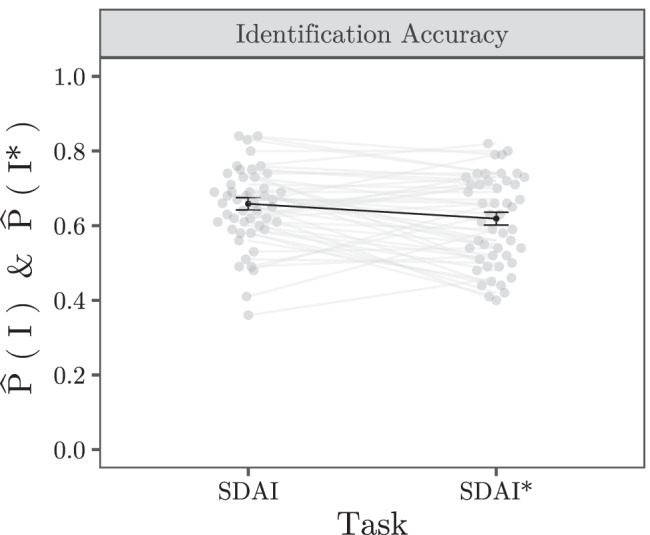
Mean relative frequencies (*black dots*) of a correct identification of an old item in the SDAI task (I) and a new item in the SDAI* task (I*). *Error bars* depict ± 1*SE* (generalized linear mixed model based), *gray dots and lines* depict individual responses (i.e., relative response frequencies of each participant)

Lastly, we compared the identification performance between the tasks by first aggregating data over participants. The rate of correct identifications was clearly lower in the m*-alternative forced-choice sub-tasks (61.10*%*) compared to the *m*-alternative forced-choice sub-tasks (64.92*%*), which was affirmed by a *χ*^2^ test (*χ*^2^(1) = 14.80, *p* < .001).^12^ To further test this effect, we also performed a generalized linear mixed model analysis of differences in identification performance between *m*-alternative forced-choice and m*-alternative forced-choice sub-tasks (using a logistic link function) where we included participants as a random-effects factor (including both by-participant random intercepts and by-participant random slopes). The result ($\chi ^{2}_{LR}(1) = 5.50$, *p* = .019; see also Fig. 6) is consistent with the result obtained from the analysis of the aggregated data.

## Discussion

The results of our investigation provide various interesting insights into the processes underlying recognition decisions. First and foremost, we see that ROC asymmetry indeed changes qualitatively when participants are tasked with detecting and identifying a new stimulus instead of an old stimulus. However, ROC asymmetry appears to be more pronounced in the SDAI task compared to the SDAI* task. This was not only indicated by the approximate zROC slopes (see Fig. 4), but also by the differences in the estimates for the old-item variance (*σ*_*o*_) between SDAI and SDAI* when fitting two separate UVGMs to the respective tasks (see Fig. 5).^13^

This observation is closely related to the finding that, if left unrestricted, the mnemonic parameters of the UVGM differed systematically between both tasks. Furthermore, restricting the mnemonic parameters of the UVGM to be independent of the task considerably degraded the model’s goodness-of-fit. This clearly indicates that even when a model is used that takes asymmetric ROCs into account, the mnemonic information underlying recognition decisions indeed changes depending on whether the tasks ask for detection and identification of an old or a new stimulus—at least when assuming normally distributed familiarity values.

However, our results allow us to draw an even stronger conclusion that does not depend on the auxiliary assumption of normally distributed familiarity values. More precisely, the fact that we observed systematic differences in correct identification rates between both the SDAI and the SDAI* task clearly speaks against the notion that the distribution of old-item familiarities remains unchanged between the two tasks. Importantly, this corroborates the idea that there is a fundamental change in the mnemonic information that is accessed by the decision-maker when the task asks for the detection of newness instead of oldness (Brainerd et al., 2021).

Taken together, these results indeed support an interpretation along the lines of fuzzy-trace theory (Brainerd et al., 2021) and defend them against a simple alternative account in terms of ROC asymmetry. In particular, uniquely identifying memory information (the so-called *verbatim trace*, which is stored only for old items) may be easier to access during the SDAI than the SDAI* task. To see why this is the case, suppose that access to verbatim traces can be modeled as a threshold process, that is, those memory traces are either accessed by the decision-maker with a certain probability, which in turn leads to a correct detection and identification response, or they are not accessed with the respective complementary probability. Let us further capture the contribution of other partial-identifying information (the so-called *gist trace*) by an EVGM. When combining both retrieval mechanisms, the resulting hybrid model is essentially equivalent to the dual-process SDT model of recognition memory (see, e.g., Yonelinas, 1994). In this hybrid model, ROC asymmetry increases with the probability of retrieving the verbatim trace (see also Pratte & Rouder, 2011). Thus, the differences between the SDAI and the SDAI* task in both the magnitude of the observed ROC asymmetry and in the identification performance can be accounted for by an impaired ability to access the verbatim traces in the SDAI* task compared to the SDAI task (i.e., when participants were asked to detect and identify the new instead of the old item), as proposed by Brainerd et al. (2021).

Yet, the description in terms of a dual-process SDT model also highlights the fact that while fuzzy trace theory may be *one possible* theoretical explanation of our findings, it is certainly not the *only* theoretical framework that is able to account for them. In essence, a simple interpretation can be provided in terms of any theory that views the unidimensional decision variable in a recognition memory task to be a joint function of multidimensional attributes. Most dual-process theories, for example, assume that a recognition decision is determined by both a *familiarity*-driven process and a *recollection*-driven process (see, e.g., Wixted & Mickes, 2010; Yonelinas, Dobbins, Szymanski, Dhaliwal, & King, 1996; Yonelinas, 1994), representing distinct contributions of item and associative/source information, respectively. Importantly, both processes may provide (partially) independent evidence regarding a prior encounter with the respective stimulus. Provided that this evidence is diagnostic, integrating it would consequently increase discriminability compared to relying on either process alone. However, decision-makers may also be able to deliberately place more weight on one dimension than the other (e.g., Migo, Montaldi, Norman, Quamme, & Mayes, 2009; Migo et al., 2014). With that in mind, it is easy to imagine that participants combine item and associative/source information when old items are defined to be the target but place more weight on item information alone when new items are defined to be the target (e.g., because low familiarity alone might be sufficient for making a detection decision for a new item, whereas the absence of recollection is not).

These theoretical considerations also have important practical implications, as they suggest that—especially in situations in which a conservative response strategy is adopted—memory performance deteriorates when participants are asked to detect newness instead of oldness. However, for situations in which erroneous decisions are particularly momentous (e.g., in the context of real-life eyewitness identification for forensic purposes), decision-makers usually tend to employ conservative response strategies. Therefore, focusing on detecting newness instead of detecting oldness should be avoided in such situations.

Lastly, the results of the present work also question the foundational evidence for ROC asymmetry provided by Kellen et al. (2021). In particular, our finding that the distribution of old-item familiarities is altered in tasks in which the new item instead of the old item is the target removes the *experimentum-crucis* status from Experiment 3 in Kellen et al. (2021). Unfortunately, this means that there is still no clear evidence for ROC asymmetry that does not rely on either confidence ratings or bias manipulations, which were critically discussed by Kellen et al. (2021). However, our results do not imply that, conversely, there is no ROC asymmetry. In fact, it is still entirely possible that the effects observed by Kellen et al. (2021) were caused by a combination of ROC asymmetry *and* changes in the accessibility of mnemonic information. These considerations clearly highlight the need for further investigations of the mechanisms responsible for ROC asymmetry.

## Footnotes

1 This parametrization also has some theoretical appeal as accumulation of independent partial memory evidence—a key aspect of some holistic memory models, such as MINERVA 2 (Hintzman, 1984)—leads asymptotically to a normal distribution due to the central limit theorem (see, e.g., Green & Swets, 1966).

2 Note that the meanings of the terms “hit” and “false alarm” differ from those described above in the context of single-item yes/no recognition tasks in that they specifically refer only to the responses in the 1-out-of-*m* detection sub-tasks, that is, correctly or falsely reporting whether or not a target (i.e., an old item) is present in the current set of stimuli.

3 Note that “hit” (“false alarm”) in the 1-out-of-*m** detection task refers to correctly (falsely) reporting the presence of a new stimulus among old stimuli. In a similar vein, a “correct identification” in the *m**-alternative forced-choice identification sub-task refers to an identification of the new stimulus.

4 For symmetric yes/no-ROCs, assuming that the same latent memory-strength distribution underlies both newness and oldness detection is equivalent to constraining the ROCs for these two tasks to be identical. If one allows them to be asymmetric, however, this is no longer the case. But since detecting oldness is in fact logically equivalent to detecting newness for a single-item yes/no recognition task (more precisely, “old” and “not-new” judgments are equivalent, as are “new” and “not-old” judgments; Brainerd et al., 2021), the yes/no ROCs depicted in Fig. 2 would overlap perfectly if one redefines a “hit” for the task where the new items are the targets to denote correctly reporting the absence of a new item, and a “false alarm” to denote incorrectly reporting the absence of a new item (provided that the distributions of memory-strength signals do not change as a function of whether the task is to detect oldness or to detect newness). However, this is not the case for the respective ROCs of the SDAI and SDAI* tasks, that is, the ROCs would not overlap in general. In essence, this is due to the fact that for each trial of an SDAI task, either *m* new items (for non-target trials) or *m* − 1 new items and one old item (for target trials) are presented, whereas for each trial of an SDAI* task either *m* old items (for non-target trials) or *m* − 1 old items and one new item (for target trials) are presented. Hence, SDAI and SDAI* (provided that *m* ≥ 2) are not logically equivalent in the same sense as detecting oldness and newness in a single-item yes/no recognition task are.

5 Note that the CDF of a continuous real-valued RV (i.e., with support ${\Omega } \subseteq \mathbb {R}$) evaluated at *λ* ∈ Ω, is equivalent to the probability that the RV will take a value less than *λ*. Note also that if multiple events are independent (such as multiple independent RVs each being smaller than *λ*), their joint probability simply equals the product of their respective individual probabilities. Since—as outlined above—all individual familiarities are assumed to be independent RVs in the present context, the joint probability that all *m* familiarity values elicited during a target trial of an SDAI task (i.e., one old-item familiarity value and *m* − 1 new-item familiarity values) fall below the response criterion *λ* is given by *F*_*o*_(*λ*)[*F*_*n*_(*λ*)]^*m*− 1^.

6 Recall that in an SDAI* task the new stimulus is considered to be the target. Thus, the meaning of “hit”, “false alarm”, and “correct identification” changes accordingly.

7 For the same reasons outlined in Footnote 5, the joint probability that all *m* familiarity values elicited during a target trial of an SDAI* task (i.e., *m* − 1 old-item familiarity values and one new-item familiarity value) exceed the response criterion *λ* is given by [1 − *F*_*o*_(*λ*)]^*m*− 1^[1 − *F*_*n*_(*λ*)].

8 This is the case, because the distribution of the effective decision variable (i.e., the maximum or minimum of all *m* normally distributed familiarities) is no longer normal.

9 The same holds true for other distributional assumptions that give rise to asymmetric ROCs as, for example, a Gumbel (minimum) distribution (Kellen & Klauer, 2018) or a Gaussian mixture distribution (DeCarlo, 2002).

10 This follows immediately from elementary probability theory, as the maximum (minimum) of a single RV is clearly just the RV itself.

11 Note that this procedure entails that participants are required to always give an identification response, even when they indicated that they believe a target to be absent (i.e., by responding ”target likely absent” or ”target definitely absent”). The reason for taking this approach was that it ensured identification responses were available for every target trial, which was necessary for the above-mentioned critical test.

12 Note that the performance level and the size of the difference are similar to the ones reported by Kellen et al. (2021, Fig. 12) for *m* ∈{4, 5, 6}.

13 An interesting side note is that these results also add to a body of evidence (Koen & Yonelinas, 2010; Koen & Yonelinas, 2013; Rouder, Pratte, & Morey, 2007; Spanton & Berry, 2020; Rabe, Lindsay, & Kliegl, 2021) that questions one popular theoretical justification for the variance difference between old-item and new-item familiarity distributions of the UVGM, namely old-item encoding variability (Jang, Mickes, & Wixted, 2012; Mickes, Wixted, & Wais, 2007; Starns, Rotello, & Ratcliff, 2010; Wixted, 2007): If processes during encoding were solely responsible for a higher variance of old-item familiarity values (compared to new-item familiarity values), this difference between variances should not depend on the type of task performed during test, as long as the conditions during encoding were identical. Contrary to this prediction, however, we observed such a dependency in the present study.
